# Supplementary material for: Oxidation-stable amine-containing adsorbents for carbon dioxide capture
Source: Nat Commun. 2018 Feb 20;9:726. doi: 10.1038/s41467-018-03123-0 (PMC5820286; doi:10.1038/s41467-018-03123-0)
Supplement: Supplementary file 1 — Supplementary Information [file 41467_2018_3123_MOESM1_ESM.pdf]

Oxidation-stable amine-containing adsorbents for carbon dioxide capture

Kyungmin Min et al.

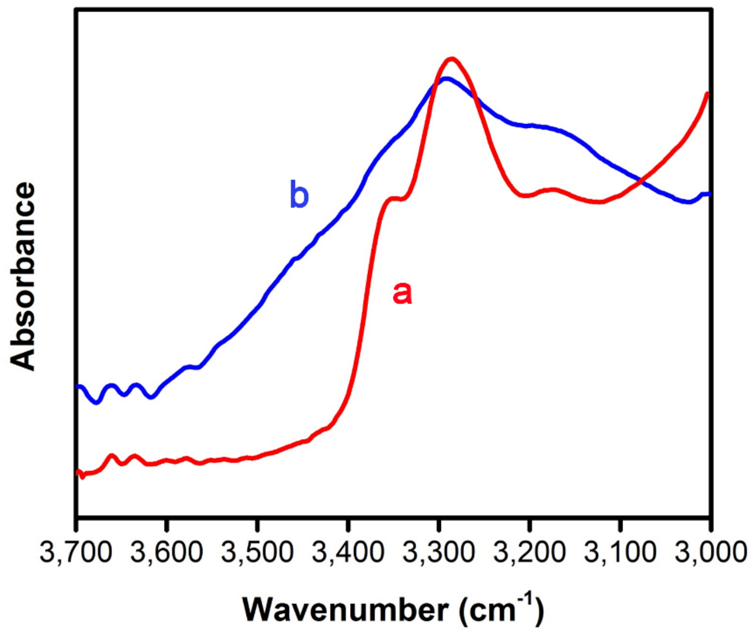

Supplementary Figure 1 FT-IR spectra of fresh adsorbents. **a-b**. PEI/SiO_2 (**a**) and EB-PEI/SiO_2 (**b**) samples.

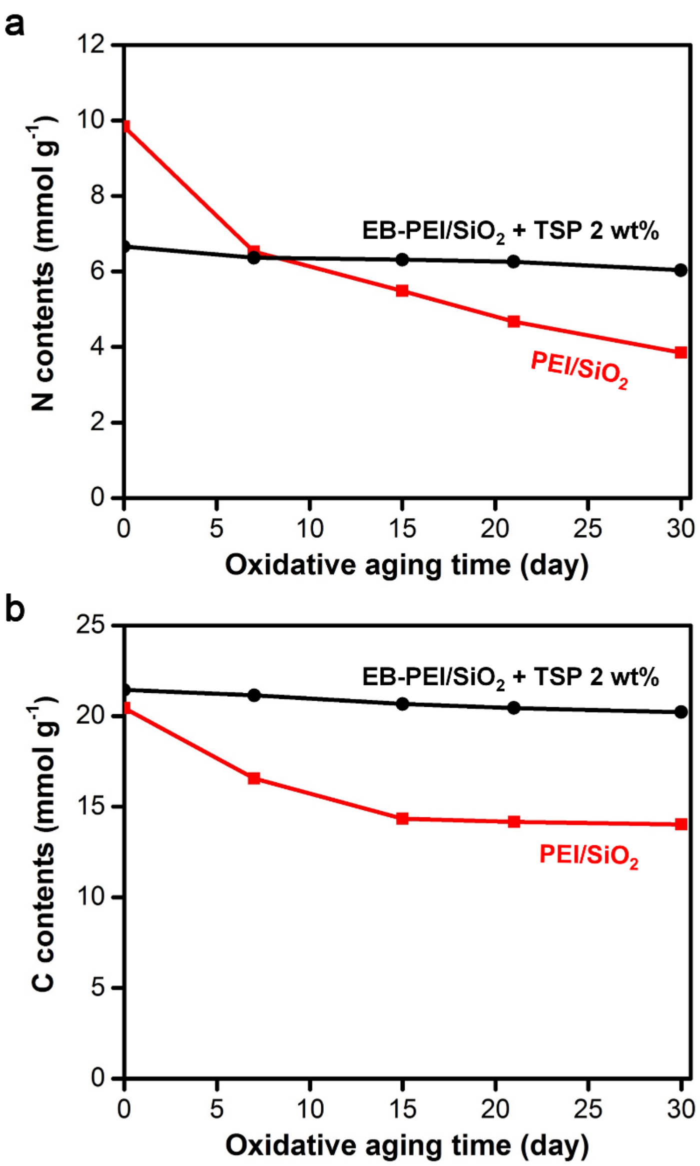

Supplementary Figure 2 Variation of nitrogen and carbon contents in adsorbents after oxidative aging. **a-b**. Nitrogen (**a**) and carbon (**b**) contents in PEI/SiO_2 without trisodiumphosphate (TSP) and EB-PEI/SiO_2 with 2 wt% TSP, before and after the oxidative aging under 3% O_2, 15% CO_2, 10% H_2O in N_2 balance at 110 °C for specified periods.

Supplementary Table 1 Contents of transition metal impurities in PEI and EB-PEI.

Species	Metal contents in PEI (ppm)[a]	Metal contents in EB-PEI (ppm)[a]
Fe	17.2	17.0
Cu	6.85	6.93
Cr	2.12	1.26
Ni	1.79	1.06

[a]The metal contents were determined by ICP-MS.

Supplementary Table 2 CO_2 working capacities of PEI/SiO_2 adsorbents containing different loadings of various chelators.

**Adsorbent**	**CO_2 working capacity (mmol g^{-1})**
PEI/SiO_2	1.98
PEI/SiO_2 + **1** (0.25 wt%)	1.97
PEI/SiO_2 + **1** (2.0 wt%)	1.96
PEI/SiO_2 + **2** (0.25 wt%)	1.97
PEI/SiO_2 + **2** (2.0 wt%)	1.95
PEI/SiO_2 + **3** (0.25 wt%)	1.96
PEI/SiO_2 + **3** (2.0 wt%)	1.95
PEI/SiO_2 + **4** (0.25 wt%)	1.97
PEI/SiO_2 + **4** (2.0 wt%)	1.96
PEI/SiO_2 + **5** (0.25 wt%)	1.97
PEI/SiO_2 + **5** (2.0 wt%)	1.95
PEI/SiO_2 + **6** (0.25 wt%)	1.96
PEI/SiO_2 + **6** (2.0 wt%)	1.95

Supplementary Table 3 CO_2 working capacities of EB-PEI/SiO_2 adsorbents containing different loadings of various chelators.

Adsorbent	CO_2 working capacity (mmol g^{-1})
EB-PEI/SiO_2	1.62
EB-PEI/SiO_2 + **1** (0.25 wt%)	1.61
EB-PEI/SiO_2 + **1** (2.0 wt%)	1.61
EB-PEI/SiO_2 + **2** (0.25 wt%)	1.61
EB-PEI/SiO_2 + **2** (2.0 wt%)	1.60
EB-PEI/SiO_2 + **3** (0.25 wt%)	1.60
EB-PEI/SiO_2 + **3** (2.0 wt%)	1.59
EB-PEI/SiO_2 + **4** (0.25 wt%)	1.60
EB-PEI/SiO_2 + **4** (2.0 wt%)	1.60
EB-PEI/SiO_2 + **5** (0.25 wt%)	1.61
EB-PEI/SiO_2 + **5** (2.0 wt%)	1.60
EB-PEI/SiO_2 + **6** (0.25 wt%)	1.60
EB-PEI/SiO_2 + **6** (2.0 wt%)	1.60
